# Gonorrhœal Arthritis and Ophthalmia Neo-Natorum

**Published:** 1903-10-24

**Authors:** 


### GONORRHŒAL ARTHRITIS AND OPHTHALMIA NEONATORUM.

That a gonococcal urethritis may be followed by a specific synovitis or arthritis is a sequence of events only too familiar. It is much less usual to observe such a development when the primary site of the disease is the conjunctiva. But a sufficient number of cases have now been recorded to show that an affection of one or more joints may complicate the gonorrhœal ophthalmia of the newly born, just as it may, and not infrequently does, follow the urethritis of a later age. The first observation of this association was made in 1885 by Mr. Clement Lucas, and the accuracy of his teaching has since been confirmed by a number of independent authorities. There is one aspect of these cases which demands attention in reference to its bearing on the treatment of the more ordinary form of gonorrhœal arthritis. We refer to the prompt and satisfactory manner in which the affected joints recover. Almost without exception, after a brief period of fixation, the joints regain their normal condition, provided—and the qualification is a significant one—the conjunctival discharge is successfully terminated. This presents a very sharp contrast to the chronic course and obstinate resistance to treatment usually seen in the gonorrhœal rheumatism of the adult. Now it cannot be that the delicate tissues of an infant are able to offer a more successful resistance to the gonococcal poison than are the tissues of the adult. The difference is rather to be found in the readiness with which the supply of the poison is cut off in the one case, and the difficulty of checking the supply in the other. The conjunctiva is readily accessible to the most thorough measures of treatment, whilst the urethra and the vagina are much less under control. Hence, in the one instance the opportunity for repeated poisoning of the blood is promptly closed, whilst in the other it exists for a relatively prolonged time. The course of the joint affections exactly corresponds to this difference. In ophthalmia neonatorum they promptly disappear, whilst the gonorrhœal rheumatism of the adult is apt to extend over many months. This contrast bears a practical lesson, namely, that the primary and imperative claim in the treatment of gonorrhœal arthritis is the adoption of the measures necessary to stop all urethral discharge. So long as this continues, so long will the opportunities for absorption of the poison and repeated damage to the joint tissues exist. In many cases, if not in the majority, the obstinate course of gonorrhœal arthritis finds its interpretation in a failure to recognise this truth.

The urethritis is either not completely cured, or wilfully or by inadvertence its existence is concealed, or the connection between it and the joint affection is not appreciated. Prompt suppression of the discharge, fixation of the affected joints, a generous diet and general tonics—these measures if adopted early and practised thoroughly render gonorrhœal arthritis an eminently curable affection.

